# Separating Wheat from Chaff in Plant Genomes

**DOI:** 10.1371/journal.pbio.0030039

**Published:** 2005-01-04

Plant genome sizes span the modest—54 million base pairs (Mb) in the bitter cress Cardamine amara—to the enormous—124,000 Mb in the lily Fritillaria assyriaca. By comparison, fruitfly and human genomes have 180 Mb and 3,200 Mb, respectively. Genomes of important crops such as sorghum, soybean, maize, and wheat hover between 735 Mb and 16,900 Mb, and determining their complete sequences is daunting and costly.

Wide size variations do not necessarily reflect differences in gene content, but rather reflect the presence of repetitive sequence elements that do not generally code for genes. Repetitive elements account for at least 75% of the maize and sorghum genomes. In a new study, Joseph Bedell and his colleagues describe a way to filter away repetitive elements when sequencing the genome of sorghum (Sorghum bicolor), a staple crop in much of the developing world because of its resilience in arid climates.

The authors use an approach known as methylation filtration that has been employed before for pilot plant genome analyses. Here they present compelling evidence of the method's reliability when applied to large-scale genome sequencing. The approach is built on the observation that in plants, methylation—a chemical tagging of DNA with methyl groups—occurs at repetitive sequences to a much greater degree than at gene sequences. This provides an opportunity to concentrate sequencing efforts on the coding portion of the genome.

To eliminate repetitive sequences, the authors introduced small pieces of sorghum chromosomes into bacteria strains designed to specifically destroy DNA sequences that carry methyl groups. Using two independent assessments, they estimated that methylation filtration reduced the amount of sorghum DNA they would need to sequence by two thirds, from 735 Mb to approximately 250 Mb.

But were any genes lost in the filtration step? The authors compared their results to partial sequence information generated previously from bacterial artificial chromosomes (BACs). BACs offer the most comprehensive representation of the genome because they contain large pieces of unmodified sorghum chromosomal DNA. Of the 148 genes identified on 14 sorghum BACs, 133 appeared in the filtered set. This means that the methylation filtration method captured at least 90% of the genes in the sorghum genome and 96% (131/137) if a repeat cluster of 11 known methylated genes is removed from the analysis.[Fig pbio-0030039-g001]

**Figure pbio-0030039-g001:**
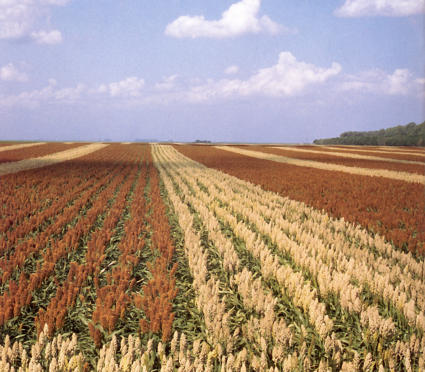
A field of hybrid sorghum

Methylation filtration also compared favorably to shotgun sequencing, a method that reads the whole genome in small fragments that are progressively assembled into larger pieces by computer analysis. The authors reported that after sequencing 285 Mb of filtered sorghum DNA—approximately 1.15 times the length of the sorghum coding regions—they obtained on average 65% of the length of 96% of the genes. Theoretical calculations and simulation based on the genome of Arabidopsis—a plant model organism—predicted that shotgun approach would yield similar results (67% of the length of 96% of the genes) after sequencing the equivalent of 1.15 times its total length (rather than 1.15 times the length of just the coding regions). Thus, methylation filtration can provide as much information on coding sequences as the shotgun approach, with less investment in sequencing.

Methylation filtration does not yield a complete genome map, but it offers quicker, more affordable access to genes than most commonly used sequencing approaches. Sorghum is closely related to maize and sugar cane, and more distantly to rice. The availability of its genome sequence offers the chance for more in-depth experiments into the evolution of the grass family, and promises important insights into the genetic control of drought resistance.

